# Assessing Early Heterogeneity in Doubling Times of the COVID-19 Epidemic across Prefectures in Mainland China, January–February, 2020

**DOI:** 10.3390/epidemiologia2010009

**Published:** 2021-03-11

**Authors:** Isaac Chun-Hai Fung, Xiaolu Zhou, Chi-Ngai Cheung, Sylvia K. Ofori, Kamalich Muniz-Rodriguez, Chi-Hin Cheung, Po-Ying Lai, Manyun Liu, Gerardo Chowell

**Affiliations:** 1 Department of Biostatistics, Epidemiology and Environmental Health Sciences, Jiann-Ping Hsu College of Public Health, Georgia Southern University, Statesboro, GA 30460, USA; so01935@georgiasouthern.edu (S.K.O.); km11200@georgiasouthern.edu (K.M.-R.); ml16842@georgiasouthern.edu (M.L.); 2 Department of Geography, Texas Christian University, Fort Worth, TX 76109, USA; xiaolu.zhou@tcu.edu; 3 Department of Psychology and Criminal Justice, School of Education & Behavioral Sciences, Middle Georgia State University, Macon, GA 31206, USA; chingai.cheung@mga.edu; 4 Independent Researcher, Hong Kong Special Administrative Region, China; westerpants@gmail.com; 5 Department of Biostatistics, Boston University, Boston, MA 02215, USA; pylai@bu.edu; 6 Department of Population Health Sciences, School of Public Health, Georgia State University, Atlanta, GA 30302, USA

**Keywords:** coronavirus, COVID-19, doubling time, epidemiology, geography, Hu Line, SARS-CoV-2, spatial analysis, spatial clustering

## Abstract

To describe the geographical heterogeneity of COVID-19 across prefectures in mainland China, we estimated doubling times from daily time series of the cumulative case count between 24 January and 24 February 2020. We analyzed the prefecture-level COVID-19 case burden using linear regression models and used the local Moran’s I to test for spatial autocorrelation and clustering. Four hundred prefectures (~98% population) had at least one COVID-19 case and 39 prefectures had zero cases by 24 February 2020. Excluding Wuhan and those prefectures where there was only one case or none, 76 (17.3% of 439) prefectures had an arithmetic mean of the epidemic doubling time <2 d. Low-population prefectures had a higher per capita cumulative incidence than high-population prefectures during the study period. An increase in population size was associated with a very small reduction in the mean doubling time (−0.012, 95% CI, −0.017, −0.006) where the cumulative case count doubled ≥3 times. Spatial analysis revealed high case count clusters in Hubei and Heilongjiang and fast epidemic growth in several metropolitan areas by mid-February 2020. Prefectures in Hubei and neighboring provinces and several metropolitan areas in coastal and northeastern China experienced rapid growth with cumulative case count doubling multiple times with a small mean doubling time.

## 1. Introduction

The coronavirus disease 2019 (COVID-19) pandemic, caused by the severe acute respiratory syndrome coronavirus 2 (SARS-CoV-2), began in Wuhan and soon spread globally. Prior studies have investigated SARS-CoV-2 transmission dynamics in mainland China [1]. For instance, population mobility data was found to be highly predictive of COVID-19 importation risk from Wuhan to other Chinese cities in early 2020 [2]; population flow from Wuhan to 296 prefectures was found to drive the spatiotemporal distribution of COVID-19 cases in China in the spring of 2020 [3]. However, detailed descriptions of mainland China’s sub-provincial administrative units that reported COVID-19 data (i.e., prefecture or equivalents) remained a gap in the epidemiological literature.

The doubling time of cumulative case count describes how fast an epidemic is growing [4]. For example, in 2003, the doubling times of the severe acute respiratory syndrome (SARS) epidemic in different countries across the world was investigated, and it was suggested that variation in doubling times of SARS epidemics across countries might arise from variation in both transmission rates and control efforts [5]. In 2020, doubling times of the COVID-19 pandemic across different European countries, different regions in Iran, and different states of the United States (U.S.) have been studied, respectively [6,7,8,9]. We previously analyzed the COVID-19 epidemic doubling time by province in mainland China, from 20 January through 9 February 2020 [10]. Further analysis by prefecture would provide a more detailed account of the early phase of the pandemic within mainland China. We hypothesized that population size or population density might be correlated with the arithmetic mean of the doubling times and ran regression models accordingly. We also investigated the power-law relationship between cumulative case count and population size. Furthermore, we investigated potential prefecture-level spatial clustering of the cumulative case count, the total times the cumulative case count doubled and the mean doubling times. Spatial autocorrelation Moran’s I statistics was performed to identify potential clusters.

The objectives of this paper are: (1) to describe the sub-provincial administrative units that reported COVID-19 case count data as reflected in an oft-cited dataset, and whether their cumulative case count in the early phase of the epidemic follows a power-law relationship with population size; (2) to compute the COVID-19 epidemic doubling times by prefecture in mainland China in the early phase of the epidemic and its relation with population size and density; and (3) to identify spatial clusters of the cumulative number of cases and the total times the cumulative case count doubled by prefecture in mainland China in the early phase of the epidemic.

## 2. Materials and Methods

### 2.1. Geographic Scope

The geographic area of this study was mainland China comprising 22 provinces, 5 ethnic minority “autonomous regions” and 4 centrally administered municipalities (Appendix A). In the provinces and “autonomous regions,” there were three tiers of sub-provincial administrative units: prefecture-level units, county-level units, and township-level units [11]. Among the prefecture-level units, the majority were prefectural-level cities that encompass cities and their surrounding counties [11]. In the centrally administered municipalities of Beijing, Chongqing, Shanghai, and Tianjin, there were two sub-municipal levels: urban districts and street communities [11]. 

### 2.2. COVID-19 Cumulative Incidence Data Sources

Sub-provincial cumulative numbers of confirmed COVID-19 cases were reported daily by provincial health commissions since 20 January 2020 [10]. Such data were collated daily by DingXiangYuan (abbreviated as DXY), an online community of mainland Chinese healthcare professionals [12]. DXY maintained a publicly available and oft-cited website that published the aggregated data that was updated daily. DXY was the source of mainland Chinese COVID-19 case count data available on the oft-cited Johns Hopkins University dashboard [13]. We downloaded DXY data from an openly available Github source that crawled data from DXY (a crawler developed by Isaac Lin, aka “BlankerL”) [14]. The dataset analyzed here covered one month immediately after the implementation of Wuhan’s cordon sanitaire on 23 January 2020: from 24 January 2020 (the first date of this dataset) through 24 February 2020 (the day this dataset was retrieved) by the date the data was retrieved from governmental press releases by DXY. We cleaned the dataset for errors and inconsistencies in data entry, as per the official press releases that our team collected from provincial government websites [10].

### 2.3. Epidemic Doubling Time

If the cumulative case count *C*(*t*) doubles between time point *t*_1_ and time point *t*_2_, i.e., *C*(*t*_2_)/*C*(*t*_1_) = 2, the time difference *t*_2_ − *t*_1_ is known as the epidemic doubling time, *t_d_*. A long epidemic doubling time indicates a slow epidemic growth. The shorter the doubling time, the faster the epidemic grows. We computed how long it took for the cumulative case count to double each time. The arithmetic means of the successive epidemic doubling times provide summaries of the epidemic growth of a location over the study period. Given that the doubling times are inversely proportional to the growth rate, and given a fixed study period, the number of times the cumulative case count has doubled provides a crude indicator of the epidemic growth; however, this is sensitive to the starting value of the cumulative incidence. The numbers of doubling times are presented alongside their arithmetic mean by prefecture.

### 2.4. Regression

We ran linear regression models with the dependent variable the arithmetic mean doubling time, and independent variable (a) population size or (b) population density, with the date of the first reported COVID-19 case as a covariate. The unit of analysis was a prefecture. For these prefectures, we obtained their population data from the 2010 China Census [15], and the geographical area data from the respective English language Wikipedia page for each province, autonomous region, or centrally administered municipality. We excluded prefectures where the cumulative case count doubled two or fewer times by 24 February 2020, from our regression models, as they introduced a lot of noise therein.

We characterized the functional relationship between population size and cumulative case count in prefectures excluding those in Hubei by 24 February 2020. Prefectures in Hubei were excluded because their cumulative case count was disproportionately high compared to those in other provinces. If the relationship between population size and cumulative case count follows the power-law, then log(cumulative case count) = *g* * log(population size), or, log(cumulative case count/population size) = *m* * log(population size) where *m* = *g* − 1. Per capita cumulative case count would be exactly proportional to population size and there would be no heterogeneity when *m* = 0. Prefectures with lower population size would have a higher per capita cumulative case count when *m* < 0; and a lower per capita cumulative case count when *m* > 0. Linear regression was used to obtain an estimate of *m* [16]. Please refer to the Appendix A for further details.

### 2.5. Spatial Clustering

In this study, the local Moran’s *I* index was used to identify the spatial clusters of COVID-19 cases and doubling times in mainland China. Using case count as an example, the local Moran’s *I* index can be expressed as:

$$I_{i}=\frac{z_{i}-\bar{z}}{\sigma^{2}}\overset{n}{\underset{j=1,j\neq i}{\sum }}w_{ij}\left(z_{j}-\bar{z}\right)$$

where *I_i_* is the local Moran’s *I* index for location *i*; *z_i_* is the cumulative number of reported cases at location *i*; $\bar{z}$ is the mean value of reported cases; *σ*^2^ is the variance of z, and *w_ij_* is the spatial weight matrix which is represented based on a distance of weighting between locations *i* and *j*. The local Moran’s *I* index can reflect the clusters of homogeneous values (e.g., high values surrounded by neighbors with high values) [17].

### 2.6. Programming

We used Python to process the cumulative incidence data and compute the COVID-19 epidemic doubling time by prefecture. R 3.5.1 to 3.6.2 (R Core Team, R Foundation for Statistical Computing, Vienna, Austria) was used in statistical analysis. ArcGIS Pro (Version 2.4.0) was used in spatial analysis and map creation.

### 2.7. Ethics

The Institution Review Board of Georgia Southern University determined that this was not defined as human subjects research under human subjects regulations (H20364).

## 3. Results and Discussion

### 3.1. Sub-Provincial COVID-19 Reporting Units

A total of 462 sub-provincial administrative entities (reporting units) reported COVID-19 data, including zero case counts (Table 1). They comprised 448 sub-provincial geographical reporting units and 14 divisions of the Xinjiang Production and Construction Corps (XPCC) [18]. The XPCC divisions were excluded from further analysis. The 448 sub-provincial geographical reporting units are referred to as “prefectures” in this paper, even though prefectures are just one type of many different reporting units (Appendix A).

Of the 448 prefectures, 39 had zero cumulative case count by 24 February 2020. Of the remaining 409 prefectures with cases, the case in Ganjiang New District was merged with the cases in the City of Nanchang for our maps and statistical analysis. Thus, a total of 408 prefectures were used in creating maps and conducting spatial analysis. Of these 408 prefectures, 8 were excluded for our statistical analysis by population and population density due to their geographic irregularities. Thus, a total of 439 prefectures (400 with cases and 39 without cases by 24 February 2020) were included in the statistical analysis (Table 1 and Table 2). In Table 3 and Table 4, we provide the descriptive statistics of the population and population density of 439 prefectures by their cumulative number of reported COVID-19 cases.

**Figure 1:**
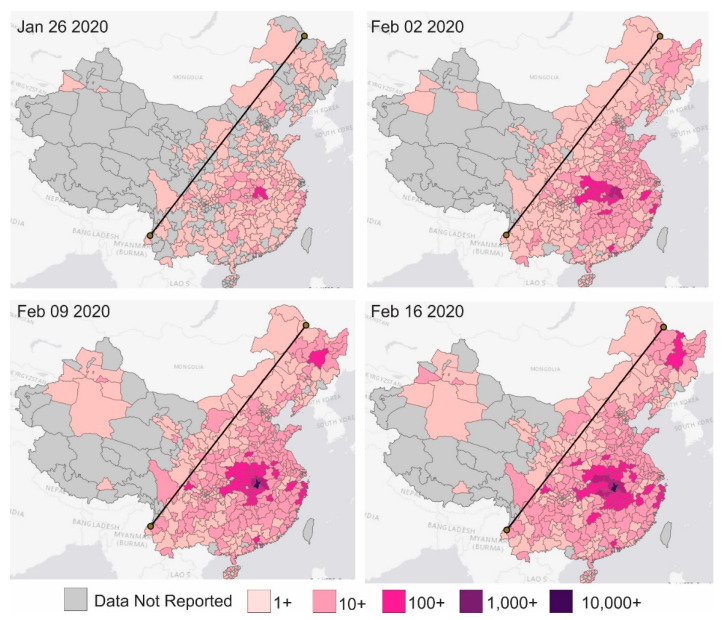
Cumulative case count by prefecture on 26 January, 2 February, 9 February, and 16 February 2020. The shade of red represents the cumulative number of cases on a log_10_ scale. The line in the plot is the Hu Line that connects the city of Heihe in Heilongjiang Province and the city of Tengchong in Yunnan Province.

**Figure 2:**
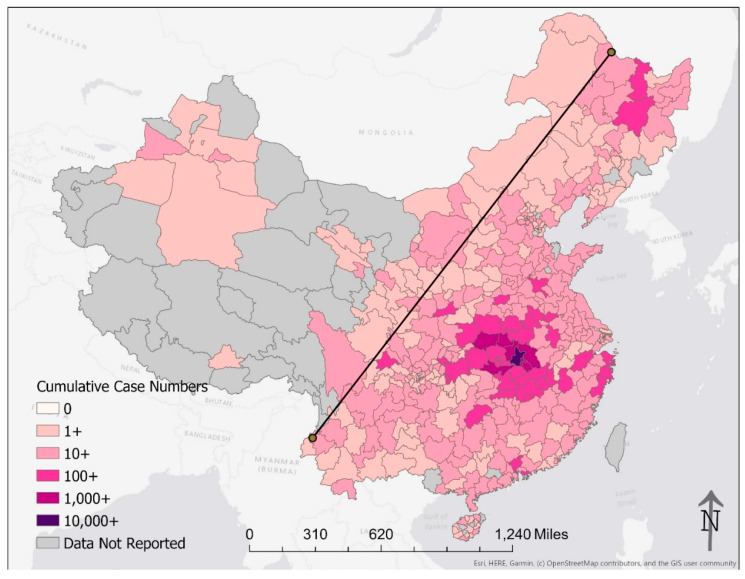
Cumulative case count by prefecture on 23 February 2020. The shade of red represents the cumulative number of cases on a log_10_ scale. The line in the plot is the Hu Line that connects the city of Heihe in Heilongjiang Province and the city of Tengchong in Yunnan Province.

Figure 1 describes the change in cumulative case count by prefecture by week from 26 January through 16 February 2020; cumulative case count on 23 February 2020, is presented in Figure 2. Our results highlight the geographic extent of the epidemic that affected many Chinese prefectures. As presented in Table 3, a total of 98% (1300 million; 2010 Census) of the mainland Chinese population lived in the 400 prefectures with at least one COVID-19 case by 24 February 2020. Nevertheless, some remote prefectures were spared. The 39 prefectures without any cases by 24 February 2020 had a population of 32 million (2%; 2010 Census). The city of Wuhan was the only reporting unit with a cumulative number of 10,000+ confirmed cases. Another seven prefectures, all in Hubei, with a total population of 27.8 million (2010 Census) had a case count in the order of thousands (1000–9999) each. Outside Hubei, all the prefectures reported a cumulative case count <1000 as of February 24. A total of 36 prefectures (8 in Hubei and 28 outside Hubei) with a total of 220 million inhabitants (2010 Census) reported case counts in the hundreds (100–999) (Table 3).

**Figure 3:**
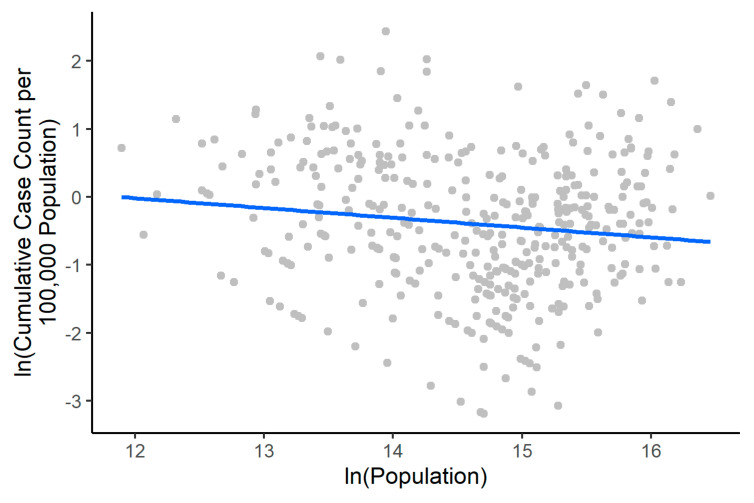
Power-law relationship between population size and cumulative case count of prefectures in mainland China (except Hubei) by 24 February 2020. The coefficient m in ln(cumulative case count per 100,000 population) = m*ln(population) is −0.1440 (95% CI, −0.2524, −0.0357).

Table 4 shows that the 39 prefectures with zero cases by 24 February 2020, had a median population density of 28.92 (2.5 to 97.5 percentiles, 0.84–514.02); the 140 prefectures with 1–9 cases had a median population density of 175.98 (9.61–25,006.65) per sq. km. The 216 prefectures with 10–99 cases had a median population density of 466.80 (59.38–22,887.07) per sq. km. The one prefecture in Hubei that fell into that group (10–99 cases) had a population density of 23.41 per sq. km and was an outlier in this group. For the 36 prefectures with 100–999 cases, the median population density was 475.62 (135.55–2143.41) per sq. km. Of these prefectures, the eight prefectures in Hubei had a median of 244.13 (137.51–529.09) and the 28 outside Hubei had a median of 550.51 (175.46–2839.21). The seven Hubei prefectures with 1000–9999 cases had a median population density of 404.57 (232.98–640.30) per sq. km and Wuhan (with 10,000+ cases) had a population density of 1144.61 per sq. km. We found that the regression line between log-transformed population size and log-transformed cumulative case count per 100,000 population (by 24 February 2020) of prefectures (excluding those in Hubei) has a slope of *m* = −0.1440 (95% CI, −0.2524, −0.0357). Given *m* < 0, low-population prefectures have a higher per capita cumulative case count than high-population prefectures (Figure 3).

**Figure 4:**
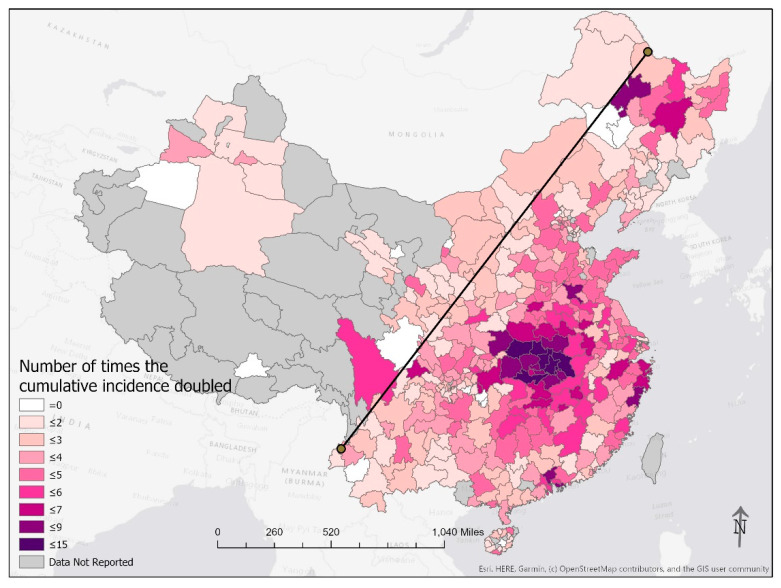
The number of times the cumulative case count doubled from 24 January through 24 February 2020. The line in the plot is the Hu Line that connects the city of Heihe in Heilongjiang Province and the city of Tengchong in Yunnan Province.

**Figure 5:**
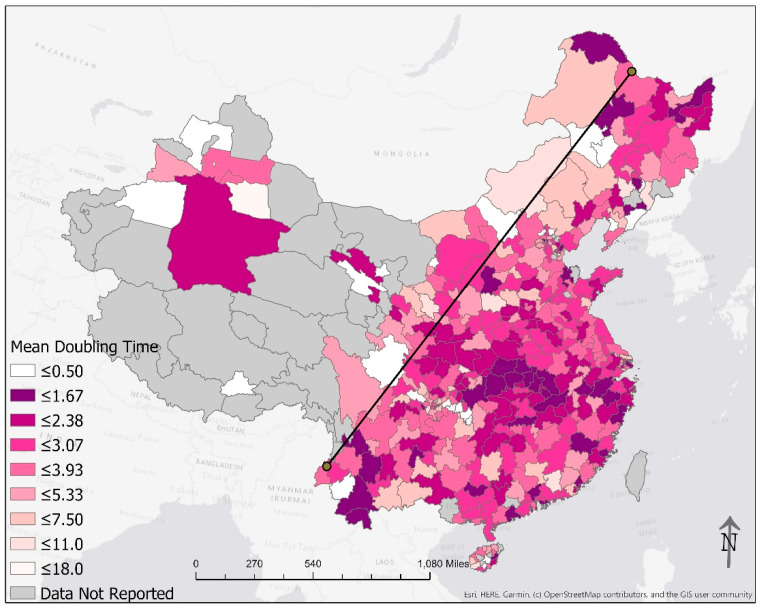
The arithmetic mean of the doubling times of the cumulative case count from 24 January through 24 February 2020. The line in the plot is the Hu Line that connects the city of Heihe in Heilongjiang Province and the city of Tengchong in Yunnan Province.

Added unto our maps is the Hu Line, a separator of population density in China first proposed by the Chinese geographer Hu Huanyong in 1935 [19]. This conceptual line is a straight line connecting Heihe, Heilongjiang Province, to Tengchong, Yunnan Province. The Hu Line divides China into a densely populated southeast and a sparsely populated northwest. The Hu Line succinctly articulates the demographic and geographic disparities in China [20]. To the northwest of the Hu Line, 6% of China’s population spans across more than half of China’s territory. That means for every square-kilometer there are 11 people, approximately one-fourth of the average global population density [21]. In contrast, approximately 94% of the Chinese population lives to the southeast of the Hu Line. It translates into a population density of 260 people per square-kilometer, approximately six times the global average [21]. By applying the Hu Line to our maps of the COVID-19 epidemic in China (Figure 1, Figure 2, Figure 4 and Figure 5), we present graphically how the vast majority of the Chinese population lived in prefectures affected by COVID-19 in February 2020. Those prefectures that reported zero cases were sparsely populated even though their areas were large.

### 3.2. Doubling Time

Figure 4 shows that the cumulative case count in prefectures in Hubei and its neighboring provinces doubled eight or more times in the study period. The cumulative case count in Wuhan doubled 15 times in the study period; another 19 prefectures doubled 8 to 11 times (Table 5). Severe epidemics happened in major metropolitan areas, such as Guangzhou and Shenzhen in Guangdong Province in the South, Wenzhou, Taizhou, Ningbo, and Hangzhou in Zhejiang Province in the East, as well as in the northeastern cities of Qiqihar and Harbin in Heilongjiang Province.

Another measure of the growth rate of the epidemic is the arithmetic mean of the doubling times (Figure 5). This metric was low in Hubei province and several coastal cities, indicating fast epidemic growth. Excluding Wuhan and excluding those prefectures where there was only one case or none, a total of 76 (17.3% of 439) prefectures had an arithmetic mean of the epidemic doubling time of <2 days (Table 5).

Among prefectures outside Hubei province and where the epidemic had doubled ≥3 times, for every increase in 100,000 residents, the arithmetic means of the doubling times changed by −0.012 (95% CI, −0.017, −0.006) after controlling for the date of the first reported case (Table 6). While the association between population size and the arithmetic mean of the doubling times was statistically significant (*p* < 0.001), the model only explained a small part of the variance (adjusted R^2^ = 0.057). We further tested if there is any association between population density and the arithmetic mean of the doubling times; the statistical association was found to be insignificant (Table 6).

### 3.3. Spatial Clustering

**Figure 6:**
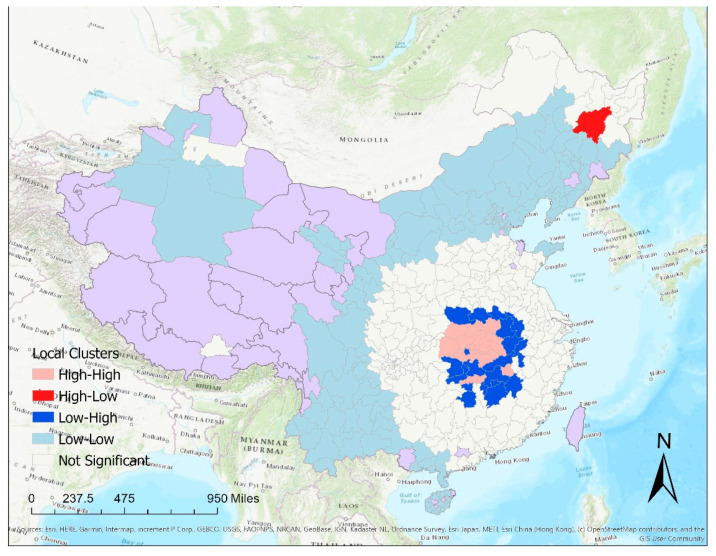
Results of local Moran’s I clusters for the cumulative number of reported cases on 16 February 2020, indicating high numbers surrounded by high numbers (high-high clusters), high numbers surrounded by low numbers (high-low clusters), low numbers surrounded by high numbers (low-high clusters) and low numbers surrounded by low numbers (low-low clusters). Purple color represents that jurisdictions without cases by 16 February 2020, or jurisdictions outside mainland China (Hong Kong, Macau, and Taiwan).

Based on the local Moran’s *I* clusters for the cumulative number of confirmed cases on 16 February 2020, most prefectures in Hubei province reported significantly more cases than prefectures in other provinces (Figure 6). Prefectures in Hubei had similarly high case numbers as their neighbors, exhibiting a high-high cluster. The prefectures adjacent to Hubei province, represented by dark blue on the map, exhibit a low-high pattern. These prefectures had significantly lower numbers than cities in Hubei province. Harbin in the Heilongjiang Province showed a high-low pattern, meaning that a significantly higher number is observed in Harbin than its neighbors.

**Figure 7:**
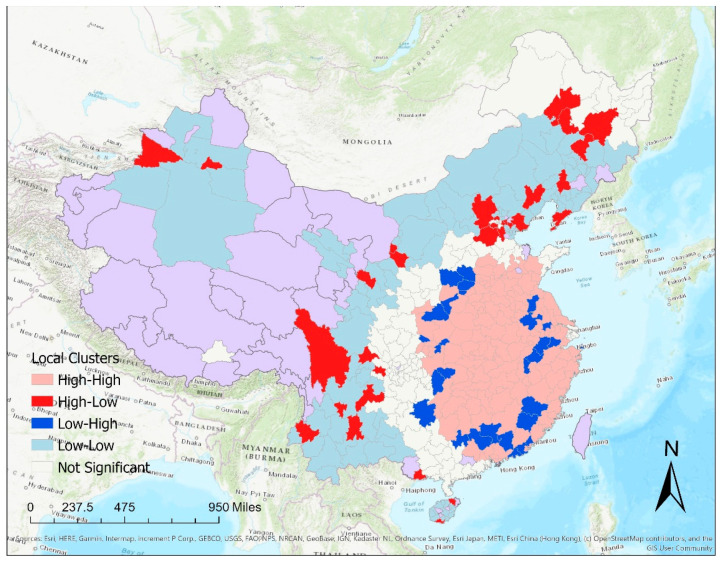
Results of local Moran’s I clusters for the total number of times the cumulative case count had doubled by 16 February 2020, indicating high numbers surrounded by high numbers (high-high clusters), high numbers surrounded by low numbers (high-low clusters), low numbers surrounded by high numbers (low-high clusters), and low numbers surrounded by low numbers (low-low clusters). Purple color represents that jurisdictions without cases by 16 February 2020, or jurisdictions outside mainland China (Hong Kong, Macau, and Taiwan).

Based on the local Moran’s *I* clusters for the total number of times the cumulative case count had doubled by 16 February 2020, many prefectures in the central and southeast part of China showed a high-high pattern, suggesting a rapid growth of the epidemic (Figure 7). A few prefectures such as Kunming, Chengdu, Baoding, and Dalian, showed significantly more times the cumulative case count doubled than their immediate neighbors are represented in a high-low pattern on the map.

**Figure 8:**
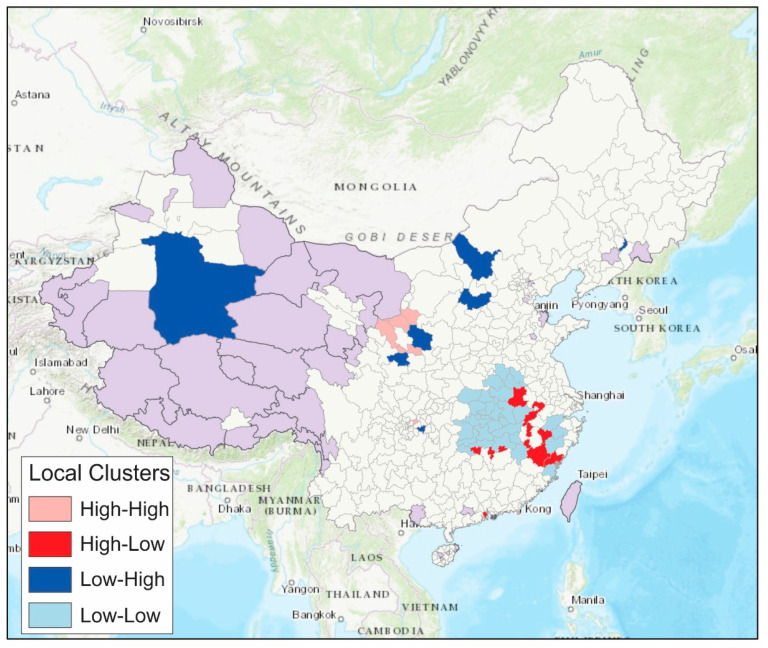
Results of local Moran’s I clusters for the arithmetic mean of the doubling times, indicating high numbers surrounded by high numbers (high-high clusters), high numbers surrounded by low numbers (high-low clusters), low numbers surrounded by high numbers (low-high clusters), and low numbers surrounded by low numbers (low-low clusters). Purple color represents that jurisdictions without cases by 16 February 2020, or jurisdictions outside mainland China (Hong Kong, Macau, and Taiwan).

Based on the local Moran’s *I* clusters for the arithmetic mean of doubling time, prefectures in the southeastern part of mainland China experienced fast epidemic growth with a short average doubling time were either in low-low clusters or high-low clusters (Figure 8). Some prefectures in the northern or northwestern part of mainland China experienced slow epidemic growth with a long average doubling time.

Our spatial clustering analysis captured a snapshot of the epidemic in mid-February 2020 when prefectures in Hubei province had very high case counts compared to the rest of mainland China. Meanwhile, prefectures across central, eastern, and southern China experienced rapid case growth. In some prefectures in northern, northeastern, and western China, epidemic hotspots were found. From the local clustering analysis, the epicenters of the epidemics can be clearly identified through the high-high clusters. Likewise, vulnerable areas located near the epicenters can be identified through the low-high clusters. Identifying the clusters of high and low case numbers can help us detect the sharpest boundaries between areas with a high and low level of transmission, which may help guide intervention measures.

If performed in real-time, spatial analysis can help epidemiologists identify epidemic hotspots and jurisdictions with rapid epidemic growth. Public health interventions can be applied in tiers that are proportionate to the risk of infection so that the epidemic can be controlled while damage to the economy and limitations to personal liberty can be minimized.

### 3.4. Limitations

This study has its limitations. First, underreporting due to underdiagnosis was a possibility, especially in Hubei province where the supply of diagnostic equipment was low and the capacity of local hospitals was overwhelmed during the study period [22]. Differential reporting rate across prefectures was a possibility too. Underlying this study was a strong assumption that the reporting rate remained the same over time within a prefecture. Given that the time frame of our dataset began on 24 January 2020, four days after the Chinese central government initiated nationwide reporting of COVID-19, we opined that the reporting rate was fairly stable during our study period. However, changes in case definitions might introduce further uncertainty in the case count reported in China as described in Tsang et al. [1]. Second, our dataset was crawled by a third-party crawler [14], from the DXY website that aggregated China’s official data for this COVID-19 pandemic. We have manually identified and corrected errors therein as per provincial governmental sources [10]. Nonetheless, some minor errors might still remain. Third, the dates in the DXY dataset were the dates that DXY retrieved the data from the health authorities. They were the same dates as the data was released as the website maintained near-real-time updates for its visitors. Since late January 2020, data every 24 h ending at midnight was released by China’s National Health Commission at around 8 am the next day (Beijing Time). Thus, the reporting date was one day behind the actual date when the cases were confirmed. Nevertheless, this limitation would not affect our calculation of the doubling times as long as the daily reporting periods remained consistent. Fourth, we acknowledged that the explanatory power of our regression models was limited because very few predictors were included in the regression models.

## 4. Conclusions

Our analysis documented spatial heterogeneity in the epidemic growth of the COVID-19 epidemic across prefectures in mainland China from 24 January to 24 February 2020. First, we found that the epidemic heavily affected prefecture-level cities in Hubei and neighboring provinces and a number of metropolitan areas in southern, eastern, and northeastern China. Nevertheless, our analysis showed that by 24 February 2020, the epidemic had spread to prefectures that comprise 98% of the Chinese population. We found that the power-law relationship between population size and cumulative case count (by 24 February 2020) indicated low-population prefectures had a higher per capita cumulative case count than high-population prefectures. Second, an increase in population size was associated with a very small reduction in the mean doubling time. Third, spatial analysis indicated that by mid-February 2020, prefecture-level cumulative case count clustered around Hubei while many prefectures across central, coastal, and northeastern China experienced rapid growth with cumulative case count doubling multiple times with a small mean doubling time. This study demonstrates that, if performed in real-time, spatial analysis of prefecture-level COVID-19 data can enable epidemiologists to stratify local jurisdictions by their epidemic growth. Tiers of public health interventions can be implemented by local jurisdictions in a manner that is proportionate to their epidemic risk. Spatial analysis can offer additional insights into the epidemic that enables effective responses to control its spread and yet minimizes unnecessary draconian measures that harm the economy and limit personal liberty.

## Figures and Tables

**Table 1:** Number and types of reporting units, DingXiangYuan (DXY) data entries by location, non-resident data entries, entries used for mapping after data merger and statistical analysis, of 31 provinces (autonomous regions, centrally administered municipalities) in mainland China.

	Number and Types of Reporting Units ^1^	DXY Data Entries (Excl. Duplicate Row)	Separate Data Entry for Non-Residents ^2^	DXY Data Entries Used for Mapping after Data Merger	DXY Data Entries Included in Statistical Analysis	Geo-Graphical Reporting Units Without Cases ^3^
Mainland China TOTAL	462	421	Not applied	408	400	39
Anhui ^4^	16 PLC	17	No	16	16	0
Beijing ^5^	16 districts	15	Yes (+1) ^2^	15	15	1
Chongqing ^6^	41 = 26 districts + 12 counties + 3 “new areas”	39	No	39	36	2
Fujian	9 PLC	9	No	9	9	0
Gansu ^7^	14 = 12 PLC + 2 AP	11	No	11	11	3
Guangdong ^8^	21 PLC	20	No	20	20	1
Guangxi ^9^	14 PLC	13	No	13	13	1
Guizhou	9 PLC	9	No	9	9	0
Hainan ^10^	19 = 4 PLC + 5 CLC + 4 counties + 6 autonomous counties	15	No	15	15	4
Hebei	11 PLC	11	No	11	11	0
Heilongjiang ^11^	13 = 12 PLC + 1 P	13	No	13	13	0
Henan ^12^	18 = 17 PLC + 1 DA CLC	23	No	18	18	0
Hubei	17 = 12 PLC + 1 AP + 3 DA CLC and 1 DA county-level forestry area	17	No	17	17	0
Hunan	14 = 13 PLC + 1 AP	14	No	14	14	0
Inner Mongolia ^13^	12 = 9 PLC + 3 leagues	11	No	11	11	1
Jiangsu	13 PLC	13	No	13	13	0
Jiangxi ^14^	12 = 11 PLC and Ganjiang New District	12	No	11	11	0
Jilin ^15^	11 = 9 PLC + 2 CLC	10	No	10	8	1
Liaoning ^16^	14 PLC	13	No	13	13	1
Ningxia ^17^	6 = 5 PLC + NECIBAC	6	No	6	5	0
Qinghai ^18^	8 = 2 PLC + 6 AP	2	No	2	2	6
Shaanxi ^19^	12 = 10 PLC + 1 CLC + YAHITZ	12	No	12	10	0
Shandong ^20^	16 PLC	15	No	15	15	1
Shanghai	16 districts	16	Yes (+1) ^2^	16	16	0
Shanxi	11 PLC	11	No	11	11	0
Sichuan	21 = 18 PLC + 3 AP	21	No	21	21	0
Tianjin ^21^	16 PLC	14	Yes (+1) ^2^	14	14	2
Tibet ^22^	7 = 6 PLC + 1 P	1	No	1	1	6
Xinjiang ^23^	14 = 4 PLC + 5 P + 5 AP	7	No	7	7	7
XPCC ^23^	14 divisions	6	No	0	0	0
Yunnan ^24^	16 = 8 PLC + 8 AP	14	No	14	14	2
Zhejiang	11 PLC	11	No	11	11	0

AP—autonomous prefectures; CLC—county-level city (cities); DA—directly administered; NECIBAC—Ningdong Energy Chemical Industry Base Administration Committee; P—prefectures; PLC—prefecture-level city (cities). XPCC—Xinjiang Production and Construction Corps; YAHTIZ—Yangling Agriculture Hi-Tech Industrial Zone. Please refer to the Appendix A for footnotes of Table 1.

**Table 2:** Reporting units excluded from statistical analysis.

Provincial-Level Units and Their Data Entries That Were Excluded (Listed in Footnotes)	Number of Entities or Data Entries Excluded ^1^	Counted towards the 462 Reporting Units	Included in the 408 Reporting Units Contributed Cases to Maps	Included in the 439 Reporting Units in the Statistical Analysis
Geographical units excluded ^1^				
Chongqing ^2^	3	Yes	Yes	No
Jiangxi ^3^	1	Yes	No	No
Jilin ^4^	2	Yes	Yes	No
Ningxia ^5^	1	Yes	Yes	No
Shaanxi ^6^	2	Yes	Yes	No
Xinjiang ^7^	14	Yes	No	No
Non-geographical units excluded				
Beijing ^8^	1	No	No	No
Shanghai ^9^	1	No	No	No
Tianjin ^10^	1	No	No	No

Please refer to the Appendix A for footnotes of Table 2.

**Table 3:** The number of reporting units, their total, median, 2.5 and 97.5 percentile population by the cumulative number of reported confirmed cases as of 24 February 2020, with population size data as per the 2010 China Census. All numbers were rounded to a whole number.

Cumulative Number of Reported Confirmed Cases as of 24 February 2020	Number of Reporting Units	Total Population	Median Population	Population at 2.5 Percentile	Population at 97.5 Percentile
**Mainland China**	**439**	**1,332,039,983**	**2,462,583**	**230,959**	**9,124,731**
0	39	31,848,609	525,570	90,714	2,447,762
1–9	140	238,294,009	1,363,741	247,335	4,341,021
10–99	216	804,313,575	3,400,676	606,085	9,036,573
100–999	36	219,989,569	6,038,972	1,114,799	12,870,158
1000–9999	7	27,808,833	4,814,542	1,215,701	6,091,515
10,000+	1	9,785,388	9,785,388	9,785,388	9,785,388
**Hubei**	**17**	**57,237,727**	**2,873,687**	**424,195**	**8,336,060**
10–99	1	76,140	76,140	76,140	76,140
100–999	8	19,567,366	2,668,135	986,318	3,933,888
1000–9999	7	27,808,833	4,814,542	1,215,701	6,091,515
10,000+	1	9,785,388	9,785,388	9,785,388	9,785,388
**Mainland China except Hubei**	**422**	**1,274,802,256**	**2,437,097**	**231,607**	**9,103,275**
0	39	31,848,609	525,570	90,714	2,447,762
1–9	140	238,294,009	1,363,741	247,335	4,341,021
10–99	215	804,237,435	3,416,196	620,985	9,039,884
100–999	28	200,422,203	7,151,485	1,425,193	13,139,293

**Table 4:** Number of reporting units, their total area (square kilometers), and the median, 2.5 and 97.5 percentile population density (number of residents per sq. km) by the cumulative number of reported confirmed cases as of 24 February 2020, with population size data as per the 2010 China Census.

Cumulative Number of Reported Confirmed Cases as of 24 February 2020	Number of Reporting Units	Total Area (Square Kilometers)	Median Population Density (Number of Residents Per sq. km)	Population Density at 2.5 Percentile (Number of Residents Per sq. km)	Population Density at 97.5 Percentile (Number of Residents Per sq. km)
**Mainland China**	**439**	**9,562,140.57**	**301.31**	**5.03**	**22,057.47**
0	39	3,183,409.11	28.92	0.84	514.02
1–9	140	3,154,046.56	175.98	9.61	25,006.65
10–99	216	2,628,555.06	466.80	59.38	22,887.07
100–999	36	511,626.98	475.62	135.55	2143.41
1000–9999	7	75,953.77	404.57	232.98	640.30
10,000+	1	8549.09	1144.61	-	-
**Hubei**	**17**	**185,824.93**	**353.20**	**68.74**	**950.00**
10–99	1	3253	23.41	-	-
100–999	8	98,069.07	244.13	137.51	529.09
1000–9999	7	75,953.77	404.57	232.98	640.30
10,000+	1	8549.09	1144.61	-	-
**Mainland China except Hubei**	**422**	**9,376,315.64**	**300.38**	**4.64**	**22,705.88**
0	39	3,183,409.11	28.92	0.84	514.02
1–9	140	3,154,046.56	175.98	9.61	25,006.65
10–99	215	2,625,302.06	468.23	61.88	22,972.87
100–999	28	413,557.91	550.51	175.46	2839.21

**Table 5:** Epidemic doubling time among 384 reporting units, excluding Wuhan (*n* = 1) and units that reported a cumulative case count of zero (*n* = 39) or one case (*n* = 15) as of 24 February 2020.

Arithmetic Mean of the Epidemic Doubling Time (Days)	Reporting Units (*n*)	% of all Geographical Reporting Units (N = 439) ^1^
0 < x < 1	13	3.0
1 ≤ x < 2	63	14.4
2 ≤ x < 3	116	26.4
3 ≤ x < 4	95	21.6
4 ≤ x < 5	44	10.0
5 ≤ x < 6	22	5.0
6 ≤ x < 7	13	3.0
7 ≤ x < 20	18	4.1
Number of times the cumulative case count doubled		
1	33	7.5
2	64	14.6
3	84	19.1
4	75	17.1
5	67	15.3
6	25	5.7
7	17	3.9
8	7	1.6
9	6	1.4
10	4	0.9
11	2	0.5

^1^ 55 (12.5%) of 439 reporting units were excluded.

**Table 6:** Coefficient estimates (95% confidence intervals) of two regression models: Model A: between the arithmetic mean of the doubling times and population size, and Model B: between the arithmetic mean of the doubling times and population density. These models were applied to data from 271 prefectures after excluding prefectures from Hubei and prefectures where the epidemic had doubled ≤2 times by 24 February 2020. Data were fitted with ordinary least squares linear regression.

Dependent Variable: Arithmetic Mean of the Doubling Times	Coefficient (95% CI)
Independent Variables	
Model A (Adjusted R^2^ = 0.057)	
Population size	−0.012 (−0.017, −0.006) *
The date of the first reported case	−0.057 (−0.124, 0.010)
Model B (Adjusted R^2^ <0.001)	
Population density	−1.65 × 10^−5^ (−4.32 × 10^−5^, 1.01 × 10^−5^)
The date of the first reported case	−1.42 × 10^−2^ (−8.13 × 10^−2^, 5.29 × 10^−2^)

* *p* < 0.001.

## Data Availability

The data used in this study was retrieved from BlankerL: DXY-COVID-19-Crawler (COVID-19/2019-nCoV Realtime Infection Crawler and API). Available online: https://github.com/BlankerL/DXY-COVID-19-Crawler (accessed on 16 April 2020).

## Appendix A

### Appendix A.1. The Scope

As a companion to our previous paper [10], the study area only covers mainland China, excluding Hong Kong, Macau, and Taiwan. The latter jurisdictions make their own public health policies that are distinct from mainland China [23] and therefore do not fall within the scope of this paper.

### Appendix A.2. Structure of Mainland China’s Local Administration

According to Chung (Chapter 1 in Chung and Lam [11]), in 2007, there were 22 provinces, 4 centrally administered municipalities, and 5 ethnic minority autonomous regions in mainland China; within which, there were 15 deputy-provincial cities, 333 prefecture-level units (283 prefecture-level cities (地級市), 20 prefectures (州), and 30 autonomous prefectures (自治州) or meng (盟, in Inner Mongolia)), 2859 county-level units (1515 counties (縣), 120 autonomous counties (自治縣), or qi (旗 in Inner Mongolia), 368 county-level cities (縣級市) and 856 urban districts (區)); and 40,813 township-level units (15,130 townships (鄉), 19,249 towns (鎮) and 6434 street communities (街道委員會)) [11]. The exact numbers would have changed since 2007. However, Chung’s chapter in Chung and Lam [11], gave us an overview of the complexity and diversity as found in the subnational and sub-provincial governance in mainland China.

### Appendix A.3. The Hu Line

The Hu Line, also known as the Hu Huanyong Line, the Heihe-Tengchong Line, or the Aihui-Tengchong Line, was a concept first proposed by Chinese geographer Hu Huanyong (胡煥庸) in 1935 [19]. This conceptual line is a straight line across mainland China, starting from Aihui County (璦琿縣) now part of the city of Heihe (黑河市) in Heilongjiang Province to the city (formerly county) of Tengchong (騰衝市) in Yunnan Province. Hu developed this concept to articulate the great disparity in geography, demography, and economic development between the southeastern part of China and the northwestern part of China. In 1935, Hu proposed that to the southeast of the Hu Line, 96% of China’s population resided in 36% of China’s landmass, while to the northwest of the Hu Line, 4% of China’s population resided in 64% of China’s landmass [19]. Even though since 1935, China’s population has increased substantially, and the territorial claims of the People’s Republic, as well as the geographical area that it effectively governs, have changed, Hu’s observation made in 1935 remains valid today: 94% of China’s population resides in 43% of China’s landmass that lies southeast to the Hu Line [21].

### Appendix A.4. The Power-Law Relationship between Cumulative Case Count and Population Size and Their Relationship with Per Capita Cumulative Case Count

As explained in the main text, if the relationship between population size and cumulative case count follows the power-law,

Cumulative case count = (population size)^*g*

log(cumulative case count) = *g* * log(population size)

which is equivalent to:

log(cumulative case count)/log(population size) = *g*

Now, the relationship between per capita cumulative case count and population size can be expressed as follows:

Per capita cumulative case count = Cumulative case count/population size

log(cumulative case count/population size) = *m* * log(population size)

log(cumulative case count) − log(population size) = *m* * log(population size)

log(cumulative case count) = (*m* + 1) * log(population size)

Therefore, *g* = *m* + 1; or, *m* = *g* − 1.

So, if we plot a figure, with log(cumulative case count / population size) on the *y*-axis and log(population size) on the *x*-axis, the slope of the regression line, i.e., *m*, would be approximately 0 (equivalent to having *g* = 1). If *m* < 0 (i.e., if *g* < 1), as population size increases, the per capita cumulative case count will decrease; if *m* > 0 (i.e., if *g* > 1), as population size increases, the per capita cumulative case count will increase.

### Appendix A.5. Footnotes to Table 1

^1^ A reporting unit in this study is defined from the perspective of epidemiological surveillance. A reporting unit is a sub-provincial unit that reports COVID-19 data to the provincial health commissions and their data are shown distinctly in a provincial health commission press release. In a province, such sub-provincial report units are usually prefecture-level cities (地級市), prefectures (州), autonomous prefecture (自治州) of ethnic minorities, or leagues (盟) in Inner Mongolia. In the four centrally administered municipalities, they are (urban) districts (區/区) and (rural) counties (縣/县).

^2^ For the centrally administered municipalities of Beijing, Shanghai, and Tianjin, the municipal government categorized non-natives to their cities as a distinct category (see Table 2). These categories were present in the DXY data set but were not included in our analysis. They were also not included in the column, “DXY data entries (excl. duplicate row)”, because these categories were not categories created by (sub-provincial) location.

^3^ These jurisdictions are not in the original DXY data set as they did not report any COVID-19 cases. They were added to the dataset for statistical analysis.

^4^ In Anhui Province, Susong County (宿松) is part of Anqing City (安庆市).

^5^ In the municipality of Beijing, Pinggu District (平谷区) did not have any cases throughout the study period.

^6^ In the municipality of Chongqing, two urban districts did not have any cases throughout the study period. The three “new areas” are Chongqing Liangjiang New Area (两江新区), Gaoxin (高新), and Wansheng (万盛经开).

^7^ In Gansu Province, three prefecture-level cities, Jiayuguan (嘉峪关市), Wuwei (武威市), Jiuquan (酒泉市) in Gansu did not report cases during the study period.

^8^ In Guangdong Province, Yunfu City (云浮市, a prefecture-level city) did not report cases during the study period.

^9^ In Guangxi Zhuang Autonomous Region, Chongzuo City (崇左市, a prefecture-level city) did not report cases during the study period.

^10^ In Hainan Province, Sansha City (三沙市, a prefecture-level city), Wuzhishan City (五指山市, a county-level city), Tunchang County (屯昌县), and Baisha Li autonomous county (白沙黎族自治县) did not report cases during the study period.

^11^ In Heilongjiang, Harbin is a sub-provincial city. It is counted as one of the 12 PLCs here.

^12^ Five subdivisions in Henan Province reported cases separately for several days. They were merged with prefecture data. Gongyi (巩义) is part of Zhengzhou City (郑州市). Hua County (滑) is part of Anyang City (安阳市). Dengzhou (邓州) is part of Nanyang City (南阳市). Yongcheng (永城) is part of Shangqiu City (商丘市). Gushi (固始) is part of Xinyang City (信阳市).

^13^ In Inner Mongolia Autonomous Region, Alxa League (阿拉善盟) did not report cases during the study period.

^14^ For Jiangxi Province, we merged the one case in Ganjiang New District (赣江新区) into Nanchang City (南昌市) for both mapping and statistical analysis, as that case was originally part of the cumulative number in Nanchang, that has been listed separately since the press release of Feb 10 on data of Feb 9.

^15^ In Jilin Province, Baishan City (白山市) did not report any cases in the study period. The two sub-prefectural-level cities that reported cases are Gongzhuling (公主岭) and Meihekuo (梅河口). They are technically parts of the prefecture-level cities of Siping and Tonghua respectively, but they reported cases under separate lines. These two sub-prefectural-level cities were excluded from the statistical analysis.

^16^ In Liaoning Province, the city of Fushun (抚顺市) did not report cases during the study period.

^17^ Technically, Ningxia Hui Autonomous Region was divided de jure into five prefecture-level cities. However, Ningdong Energy Chemical Industry Base (宁东能源化工基地) was administered under a special arrangement and the confirmed case in the township of Ningdong (宁东镇) in that Industry Base was reported under a separate line on the press release of Jan 30. The Ningdong Energy Chemical Industry Base is excluded from statistical analysis.

^18^ In Qinghai Province, only Xining City (西宁市) and Haibei Tibetan Autonomous Prefecture (海北藏族自治州) reported cases during the study period. The rest of Qinghai did not report any cases.

^19^ In Shaanxi Province, the city of Hancheng (韩城市) is a sub-prefectural county-level city that is de jure part of Weinan City but its one case was listed separately. Yangling Agriculture Hi-Tech Industrial Zone (杨凌农业高新技术产业示范区) is de jure part of Xianyang, but it is directly administered by the province; its one case was listed separately. Both Hancheng and Yangling were excluded from the statistical analysis.

^20^ In Shandong Province, the city of Dongying (东营市) did not report any cases in the study period.

^21^ In the municipality of Tianjin, Jinghai District (静海区) and Jizhou District (蓟州区) did not report any cases during the study period.

^22^ In Tibet Autonomous Region, only the city of Lhasa (拉萨市) reported one imported case from Wuhan on 29 January 2020. The other six reporting units reported zero cases.

^23^ In Xinjiang Uighur Autonomous Region, alongside the regular sub-provincial administrative units, there are settlements ran by the Xinjiang Production and Construction Corps (XPCC, 新疆生产建设兵团) [18]. Such settlements had their own organizations distinct from the rest of Xinjiang. The XPCC administration has a unique political status in China. For example, in the daily press release made by the National Health Commission on the daily case count of COVID-19, it begins as follows: “[Date] 0–24 h, 31 provinces (autonomous regions, centrally administered municipalities) and the Xinjiang Production and Construction Corps reported [number] new confirmed cases, ….” The XPCC is listed alongside the provinces. We are unable to map the XPCC settlements and only seven cities, prefectures, and autonomous prefectures were mapped even though we kept the 13 rows of data. In Xinjiang, two cities (Ürümqi 乌鲁木齐市and Turpan 吐鲁番市), two prefectures (Aksu 阿克苏地区and Tacheng 塔城地区), three autonomous prefectures (Changji 昌吉回族自治州, Bayingolin 巴音郭楞蒙古自治州and Ili 伊犁哈萨克自治州) reported cases; the other seven did not. Six of the 14 XPCC divisions (i.e., 4th, 6th, 7th, 8th, 9th, and 12th) reported cases, but they were not mapped in this paper.

^24^ In Yunnan Province, Nujiang Lisu Autonomous Prefecture (怒江傈僳族自治州) and Diqing Tibetan Autonomous Prefecture (迪庆藏族自治州) did not report any cases during the study period.

### Appendix A.6. Footnotes to Table 2

^1^ The number of sub-provincial geographical units analyzed in statistical analysis = 462 − (3 + 1 + 2 + 1 + 2 + 14) = 439. ^2^ The three “new areas” are Chongqing Liangjiang New Area (两江新区), Gaoxin (高新), and Wansheng (万盛经开). ^3^ Ganjiang New District (赣江新区). ^4^ Gongzhuling (公主岭); Meihekou (梅河口). ^5^ Ningdong Management Committee (宁东管委会). ^6^ Yangling (杨凌); Hancheng (韩城). ^7^ The 14 divisions of Xinjiang Production and Construction Corps (XPCC, 新疆生产建设兵团). ^8^ Non-resident visitors to Beijing (外地来京人员). ^9^ Non-resident visitors to Shanghai (外地来沪人员). ^10^ Non-resident visitors to Tianjin (外地来津人员).
